# Childhood trauma, substance use and mental health: Exploring differences across two Tunisian jails

**DOI:** 10.1371/journal.pone.0353367

**Published:** 2026-07-09

**Authors:** Aya Ajmi Blout, Imen Mlouki, Emna Hariz, Mariem Meherzi, Abla Chefai, Mariem Triki, Marwa Boussaid, Helen Snooks, Abir Aouissaoui, Sana El Mhamdi

**Affiliations:** 1 Department of Preventive and Community Medicine, University Hospital Tahar Sfar, Mahdia, Tunisia; 2 Epidemiology Applied to Maternal and Child Health Research Laboratory 12SP17, Monastir, Tunisia; 3 Department of Community Medicine, Faculty of Medicine of Monastir, Monastir, Tunisia; 4 Department of Forensic Medicine, Faculty of Medicine of Monastir, Monastir, Tunisia; 5 Medical School, Swansea University, Wales, United Kingdom; University of Foggia: Universita degli Studi di Foggia, ITALY

## Abstract

**Introduction:**

The increasing rate of incarceration represents a significant public health issue worldwide. However, focusing on mental health issues and exploring gender differences among this vulnerable population is lacking in the literature. We aimed to assess differences in childhood trauma, substance use, and mental health among two Tunisian jails.

**Materials and methods:**

Two cross-sectional studies were conducted in two Tunisian prisons with different gender compositions, one involving male prisoners in April 2023 and the other involving female prisoners in July 2024. We used Arabic-validated versions of the Adverse Childhood Experiences-International Questionnaire, the Hospital Anxiety and Depression Scale, and the Rosenberg Self-Esteem Scale. Substance use and suicidal thoughts were assessed through a yes or no question. The questionnaire was anonymous and participation was voluntary.

**Results:**

A total of 568 prisoners answered the questionnaire. The majority were males (71%) with a median age of 31 years [2640]. We found that participants from female prison reported higher rates of childhood sexual abuse (25.6% vs 7.8%, p < 0.001). Physical abuse was more common among the male prison population (74.6% vs 58.8%, p < 0.001). Community and collective childhood violence were significantly more prevalent among participants from the male prison (94.8% vs 74.5%, p < 0.001 and 57.3% vs 43.6%, p = 0.003, respectively). Substance use was more prevalent among participants in the male prison across all substance types assessed. The female prison population reported more severe anxiety symptoms (69.1% vs 23.6%, p < 0.001) and depressive symptoms (96.4% vs 80.1%, p < 0.001). About 46% of participants in the female prison reported suicidal thoughts during incarceration, compared with 35.1% in the male prison (p = 0.014). The male prison population was more likely to have lower self-esteem (87.4% vs 69.1%, p < 0.001).

**Conclusion:**

Implementing rehabilitation programs for prisoners is essential to reduce incarceration rates and mitigate these alarming negative outcomes.

## Introduction

The increasing rate of incarceration represents a significant public health issue worldwide [1]. According to the World Prison Brief, the United States (U.S.) had one of the highest incarceration rate, with 531 individuals per 100,000 of the national population in 2021 with a predominance of males [2]. However, recent global data reveal that the number of female prisoners is growing much faster than that of male prisoners [3]. According to the Prison Policy Initiative, the total U.S. government spending on public jails is $80.7 billion [4]. Incarcerated individuals are more susceptible to developing mental health problems and poor physical health as they are more exposed to risky behaviors, infectious and chronic diseases compared to the general population [5,6]. This can also be explained by the fact that incarceration hinders access to essential services such as healthcare, housing and employment, creating challenges for reintegration into society and recidivism [7]. Tunisia is currently ranked 77th worldwide in terms of prison population rate, with 196 people per 100,000, just 3.3% of them females [2,3]. In 2021, the occupancy level of Tunisian jails was 126.4%, revealing the prison overcrowding [8]. An understanding of contributors to criminal involvement, incarceration and differences by gender is needed, particularly in the Africa and Middle East regions, where few published data are available about this vulnerable population.

Adverse childhood experiences (ACEs) are defined by the World Health Organization (WHO) as traumatic familial or social events that occur in childhood until the age of 17 years old [9]. Several studies worldwide showed the association between ACEs and criminal behaviors with a dose response effect [10–12]. In fact, as the ACE scores increased, the risk of criminality such as active violence increased. This link can differ according to gender due to biological, psychological, cultural and economic distinctions between males and females.

One possible explanation is that stress affects the frontal cortex differently in males and females, resulting in varied responses to stress in the glutamate, Gamma-aminobutyric acid (GABA), and Brain-derived neurotrophic factor (BDNF) systems [13]. According to our knowledge, few studies have focused on explaining this association by gender among prisoners. Understanding these nuances is important for developing effective interventions for both genders, particularly in the context of reducing incarceration rates.

A further contributing factor to criminal behaviors is substance use. This addictive behavior is common among prisoners compared to the general population [6,12]. In addition, incarceration can lead to involvement in substance use [14]. Thus, substance use marginally predicted an increased rate of recidivism at one wave, and recidivism is associated with an increased rate of substance use at another [15]. In Tunisia, alarming substance use rates were recently reported in the general population, with 24.8% of adolescents consuming tobacco and 8% reporting alcohol consumption in 2021 according to Medspad III [16]. In addition, crimes were related to drug use, with almost five thousand drug traffickers arrested in Tunisia in 2024 [17]. However, too little attention has been paid to focusing on substance use in Tunisian prisoners. Exploring this according to gender is also lacking. In fact, the disparity in physiologic effects of these substances by gender may be attributed to differences in pharmacokinetic properties and gonadal hormones [18]. For example, nicotine plasma concentrations are higher in females than in males reaching peak levels more quickly [18].

Focusing on assessing mental health in this vulnerable population is crucial to understanding the path leading to criminal behaviors to implement specific and effective preventive strategies. Compared to the general population, the prevalence of depression, anxiety, suicidal ideation and sleep disorders are more common among prisoners [19]. This can be related to the harmful effects of confinement, which can be both threatening and mentally draining for prisoners. According to a recent review, the prevalence of severe mental illness and substance misuse were higher among prisoners in low- and middle-income countries [20]. However, according to our knowledge, there have been limited publications about the mental health of prisoners in these countries including Tunisia.

Given these data, this research aimed to assess differences in childhood trauma, substance use and mental health issues among adults in two Tunisian jails.

## Materials and methods

### Study design, setting and sampling

We performed two cross-sectional studies in two Tunisian prisons with different gender compositions, one involving male prisoners in April 2023 and the other involving female prisoners in July 2024. The two prisons accommodate individuals convicted of a wide range of offenses, from minor crimes to more serious offenses.

The male prison population was enrolled in the civil prison of Mahdia. This jail has a design capacity of 900 prisoners. During data collection, 1700 individuals were incarcerated. This prison was divided into two sectors: the closed correctional facility and the center for open rehabilitation where prisoners participate in activities like agriculture, carpentry and sewing.

The female prison population was enrolled in the civil prison of Messadine (Sousse) with a design capacity of 250 prisoners and overcrowded cells. Activities like cooking, sewing and literacy courses are also available in this jail.

**Fig 1:**
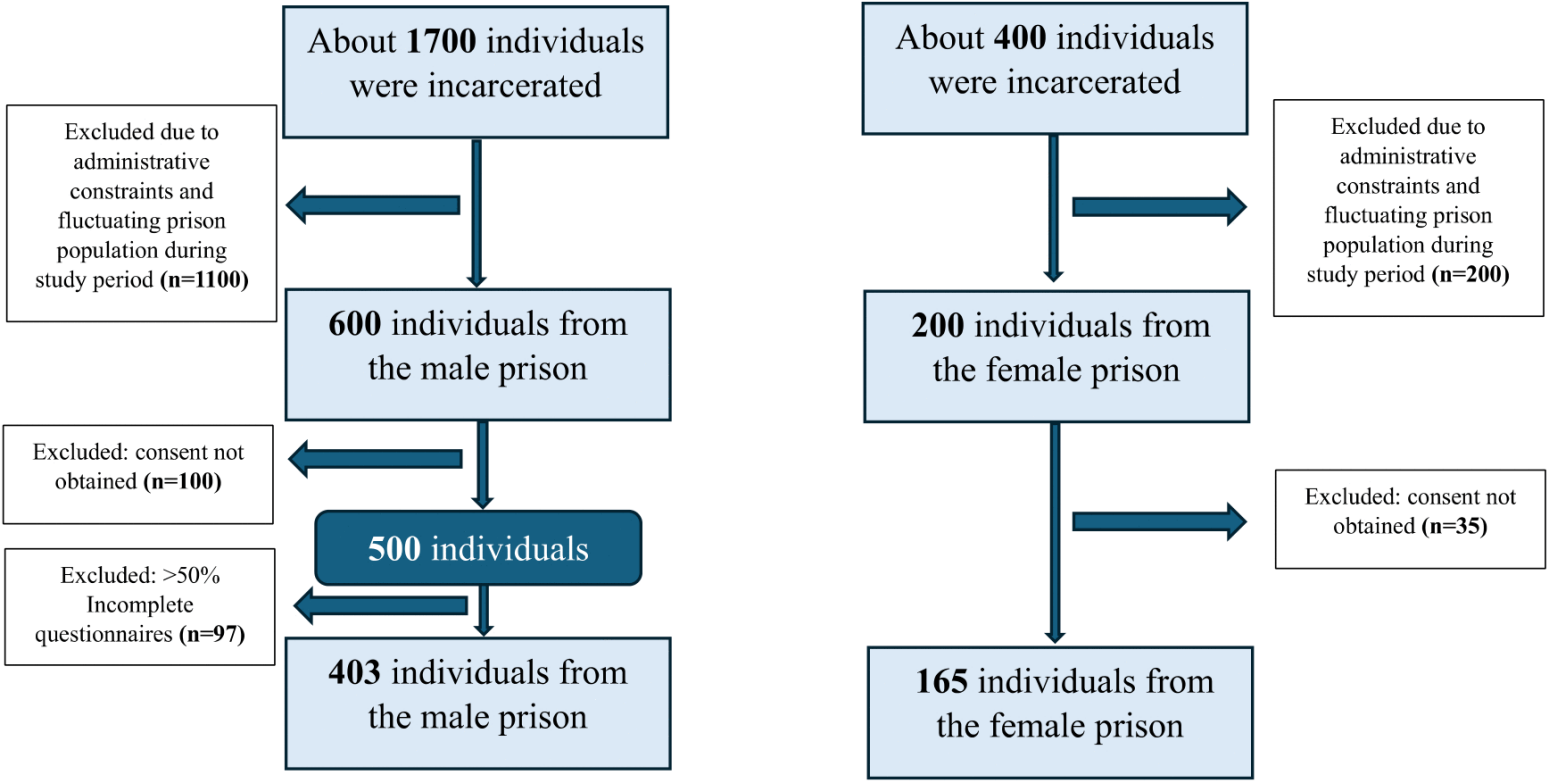
Flowchart showing the selection and exclusion of adult prisoners in the two Tunisian jails.

All prisoners were informed by the prison staff about the purpose of the study, and those who agreed to participate were admitted to the data collection room. The research team explained the study details and obtained consent. All prisoners who agreed to complete the questionnaire and were older than 18 years were included in the current survey (Fig 1.). Questionnaires with more than 50% missing responses were considered incomplete and excluded from the analysis.

The minimal size required for the study was 385 prisoners based on 0.05 probability of type one error (α), an accuracy of 5%, and a frequency of child abuse among incarcerated individuals of 49% according to literature [21].

### Data collection and study instruments

For both studies, the same doctors from the Department of Preventive and Community Medicine performed data collection using the same measurement tool. It was in Arabic, anonymous and self-administered. To ensure privacy, data collection was conducted by groups of 15–20 prisoners in a wide room with distant individual tables. Two trained doctors explained the purpose of the study to participants, emphasizing that the research was conducted solely for scientific purposes and would have no impact on their legal status, detention conditions, or access to services. Participants were also informed that access to the data was restricted to the research investigators only. Furthermore, participants were informed that the investigators belonged to an independent medical team and were not affiliated with either the prison administration or the prison healthcare staff. Then the investigator doctor clarified each item mentioned in the questionnaire. For illiterate prisoners or those having difficulties with reading and/or comprehension, filling the questionnaire was assisted by an investigator far from the others in the back of the room, and each participant was interviewed individually. Also, participants were explicitly informed that responses would remain strictly confidential and anonymized.

### Measures and tools

#### Childhood adversities.

We used the validated Arabic version of **Adverse Childhood Experiences-International Questionnaire** (**ACE-IQ**) [22]. The ACE-IQ contains two categories:

-Social ACES, including three dimensions, being exposure to peer (one item), collective (one item) and community violence (three items) during childhood.

-Intra-familial ACES including 6 dimensions being, abuse (physical, emotional and sexual; five items), neglect (physical and emotional; five items) and household dysfunction (four items).

To calculate an ACE total score, we assigned zero for “never” and one for “many times” or “few times”. For each ACE dimension, we summed its items [23] and assigned zero for no exposure and one for at least one exposure. For each ACE dimension, item responses were summed and subsequently dichotomized to indicate the absence (zero) or presence (one) of exposure, defined as endorsement of at least one item within the dimension.

A cumulative ACE score was then calculated by summing the nine dichotomized ACE dimensions, yielding a total score ranging from zero to nine, with missing data excluded from analysis.

#### Substance use among prisoners.

We asked participants three yes or no questions about substance use including current smoking, drinking alcohol before incarceration and a history of drug consumption. To assess nicotine dependence, we used the shorter form of **The Fagerström Test for Nicotine Dependence (FTND)** [24,25]. A nicotine dependence score between zero and three reflected low to moderate dependence. Otherwise, a score greater than or equal to four reflected high dependence on nicotine.

#### Mental health issues among prisoners.

The validated Arabic version of **the Hospital Anxiety Depression Scale (HADS)** was used to screen anxiety and depression during incarceration [26]. Anxiety and depression were analyzed separately. For each subscale, a total score of 11 or higher indicates significant symptoms and a total score ranging from eight to 10 is considered a doubtful case. In contrast, a score of seven or lower is considered within the normal range.

Participants were asked if they had suicidal thoughts or self-harm behaviors during incarceration through yes or no questions. We also investigated the forms of self-harm including self-mutilation, self-immolation, self-starving, self-hitting, and intentional medication overdose.

Sleep disorders were screened using the validated Arabic version of **The Pittsburgh Sleep Quality Index (PSQI)** [27]**.** A global PSQI score of five or lower indicates good sleep quality, while scores above five indicate poor sleep quality, reflecting significant sleep disturbances.

The validated Arabic version of the **Rosenberg Self-Esteem Scale (RSE)** was used to assess self-esteem among prisoners [28]. A total score of 20 or less indicates low self-esteem. Participants with scores between 21 and 23 are considered to be within the normal range, with a score between 24 and 29 indicating high self-esteem.

#### Criminological variables.

Participants were asked about the age of the first incarceration and about how many times they had been incarcerated. An individual was classified as a recidivist if they had been imprisoned more than one time. We also investigated the reason for incarceration and then the type of crime.

Types of crime were classified into three categories according to the Tunisian penal code [29].

**Felony** is a serious criminal offense that carries a penalty of imprisonment for more than five years.

**Misdemeanor offenses** are less serious crimes that can result in an imprisonment period between 16 days and five years.

**Infractions** are minor offenses usually punishable by a fine and may result in a penalty of up to 15 days.

### Statistical analysis

We used Statistical Package for the Social Sciences (SPSS) version 21 to analyze the data.

To describe qualitative variables, we used frequencies and percentages. To represent quantitative variables, we present means and standard deviations (SD) when the distribution was normal, and median and interquartile range (IQR) when it was non-normal.

To assess differences between the two populations with different gender compositions across the qualitative variables ACEs, substance use, and mental health, we performed a chi-square test. A Student t-test was conducted to examine differences in ACE scores. A Mann-Whitney U test was performed to examine differences in the age at first incarceration.

Key outcomes (ACEs, mental health issues, and substance use) were examined using multivariable binary logistic regression models, adjusting for relevant covariates.

No formal correction for multiple testing was applied.

A p-value less than 0.05 was the cut-off to consider a difference as statistically significant.

### Ethical considerations

The study protocol was approved by the Ethics Committee at the University Hospital of Mahdia (the approval number: CEM-2023-02-3).

The authorization was obtained from the Central Administration of the General Authority for Prisons and Rehabilitation, via the prison director who was provided with a copy of the questionnaire. The participation was voluntary. Incarcerated individuals were informed that they could decline or withdraw from participation at any time without any consequences or disadvantages. The questionnaire was anonymous. We obtained informed consent. Participants were provided with complete information regarding the purpose of the study and explicitly informed that responses would remain strictly confidential and anonymized. Each participant signs their own questionnaire to confirm consent, without providing any personal identifying information with the signature. Minors were not included in the study.

## Results

A total of 568 prisoners completed the questionnaire. The majority were males (71%) with a sex ratio of 2.4. Among them, 94.5% had Tunisian nationality. The participant median age was 31 years [2640].

### Childhood abuse experiences among adult prisoners

The average total ACE score was significantly higher among participants from the male prison (4.98 ± 1.94 vs 4.5 ± 2.48, p = 0.03). In fact, experiencing at least one intra-familial ACEs was two times more prevalent among this population. They were five times more likely to experience at least one social ACE (Table 1).

**Table 1:** Distribution of childhood traumatic events between two populations with different gender compositions.

	Males n (%)	Females n (%)	p-value	Odds-ratio (OR)	Odds-ratio adjusted (ORa)*	95% Confidence Interval adjusted (ICa)*
Total Intra-familial ACEs (n = 530) Yes No	341 (**92.5%)** 26 (7.1%)	138 (84.7%) 25 (15.3%)	**p = 0.003**	**2.37**	**2.13**	**[1.14-3.97]**
Emotional Abuse (n = 550) Yes No	281 (73%) 104 (27%)	115 (69.7%) 50 (30.3%)	p = 0.431	_		_
Physical Abuse (n = 547) Yes No	285 (**74.6%**) 97 (25.4%)	97 (58.8%) 68 (41.2%)	**p < 0.001**	**2.06**	**1.95**	**[1.29-2.95]**
Physical Neglect (n = 557) Yes No	154 (39.2%) 239 (60.8%)	64 (39%) 100 (61%)	p = 0.972	_		_
Household Dysfunction (n = 562) Yes No	268 (67.5%) 129 (32.5%)	107 (64.8%) 58 (35.2%)	p = 0.543	_		_
Sexual Abuse (n = 550) Yes No	30 (7.8%) 356 (92.2%)	42 (**25.6%**) 122 (74.4%)	**p < 0.001**	**4.08**	**5.54**	**[3.12-9.85]**
Emotional Neglect (n = 568) Yes No	124 (30.8%) 279 (69.2%)	44 (26.7%) 121 (73.3%)	p = 0.331	_		_
Total Social ACEs (n = 563) Yes No	391 (**97.3%)** 11 (2.7%)	139 (86.3%) 22 (13.7%)	**p < 0.001**	**5.62**	**5.52**	**[2.45-12.43]**
Bullying (n = 568) Yes No	185 (45.9%) 218 (54.1%)	83 (50.3%) 82 (49.7%)	p = 0.340	_		_
Community Violence (n = 563) Yes No	381 (**94.8%)** 21 (5.2%)	120 (74.5%) 41 (25.5%)	**p < 0.001**	**6.19**	**6.35**	**[3.33-12.09]**
Collective Violence (n = 568) Yes No	231 **(57.3%)** 172 (42.7%)	72 (43.6%) 93 (56.4%)	**p = 0.003**	**1.73**	**1.52**	**[1.03-2.24]**

*Adjustment for age, educational level, marital status, recidivism and gender.

### Substance use among adult prisoners

Table 2 summarizes the substance consumption between two populations with different gender compositions.

**Table 2:** Distribution of substance use between two populations with different gender compositions.

	Males n (%)	Females n (%)	p-value	OR	ORa*	ICa*
Tobacco use (n = 568) Yes No	310 (**76.9%)** 93 (23.1%)	92 (55.8%) 73 (44.2%)	**<0.001**	**2.64**	**2.35**	**[1.55-3.57]**
Alcohol Consumption (n = 567) Yes No	238 (**59.1%)** 165 (40.9%)	56 (34.1%) 108 (65.9%)	**<0.001**	**2.78**	**2.48**	**[1.64-3.76]**
Drug Consumption (n = 567) Yes No	210 (**52.1%)** 193 (47.9%)	57 (34.8%) 107 (65.2%)	**<0.001**	**2.04**	**1.71**	**[1.1-2.67]**

*Adjustment for age, educational level, marital status, recidivism and gender.

Participants from male prison were more likely to consume all types of substances.

The high nicotine dependence was significantly more prevalent among participants from the female prison compared to the other prison population (72.2% vs 55.6%, p = 0.019).

### Mental health issues among adult prisoners

#### Prevalence of anxiety symptoms.

Anxiety symptoms varied across the two prison populations studied. Anxiety symptoms were nearly five times more common in participants recruited from the female prison compared to the male prison population, with a confidence interval of [2.21–12.78]. Participants from the Female prison suffered more from severe anxiety symptoms (69.1% vs 23.6%, p < 0.001). On the other hand, mild and moderate anxiety symptoms were more common among individuals from the male prison than the female prison population (24.1% vs 4.2%, p < 0.001 and 35.6% vs 23%, p = 0.004, respectively).

#### Prevalence of depressive symptoms.

Depressive symptoms varied across the two prison populations studied. The prison population composed of females showed higher odds of depressive symptoms than the population composed of male prisoners, with an odds ratio of 7.03 and a confidence interval ranging from 2.94 to 16.81. Moderate to severe depressive symptoms were more prevalent in the female prison population (96.4% vs. 80.1%, p < 0.001), whereas mild depressive symptoms were more prevalent in the male prison population (22.6% vs. 10.9%, p = 0.001).

#### Prevalence of suicidal thoughts and self-harming.

About 46% of the female prison population reported experiencing suicidal thoughts during incarceration, compared to 35.1% of individuals from the male prison (p = 0.014). Self-harm was reported by 46% of participants from the male prison and 43% of those from the female prison (p = 0.5).

#### Types of self-harming behaviors.

Individuals from the female prison were statistically more likely to self-harm through self-starving (75.4% vs 28.1%, p < 0.001), self-hitting (29.4% vs 16.6%, p = 0.012) and intentional medication overdose (31.9% vs 15.9%, p = 0.002). See more details in Table 3.

**Table 3:** Distribution of reported suicidal thoughts and self-harming behaviors between two populations with different gender compositions.

	Males n (%)	Females n (%)	p-value	OR	ORa*	ICadjusted*
Suicidal thoughts (n = 567) Yes No	141(35.1%) 261(64.9%)	76**(46.1%)** 89(53.9%)	**0.014**	**1.581**	**1.765**	**[1.186-2.626]**
Self-Harming (n = 566) Yes No	184(45.9%) 217(54.1%)	71(43%) 94(57%)	0.535	_		_
Self-mutilation (n = 459) Yes No	115 (29.5%) 275 (70.5%)	20 (29.5%) 49 (71%)	0.933	_		_
Self-immolation (n = 460) Yes No	29 (7.4%) 362 (92.6%)	10 (14.5%) 59 (85.5%)	0.052	_		_
Self-starving (n = 361) Yes No	11 (28.1%) 281 (71.9%)	52 **(75.4%)** 17 (24.6%)	**<0.001**	**7.81**	**8.098**	**[4.41-14.82]**
Self-hitting (n = 459) Yes No	65 (16.6%) 326 (83.4%)	20 **(29.4%)** 48 (70.6%)	**0.012**	**2.1**	**2.24**	**[1.22-4.10]**
Intentional medication overdose (n = 459) Yes No	62 (15.9%) 328 (84.1%)	22 **(31.9%)** 47 (68.1%)	**0.002**	**2.47**	**2.74**	**[1.50-4.99]**

*Adjustment for age, educational level, marital status, recidivism and gender.

#### Prevalence of sleeping disorders.

The majority of participants were identified with poor sleep quality (90.6%). After adjusting for age, education, marital status, recidivism, and gender, individuals from the female prison were more likely to have poor sleep quality (93.20% vs 84.7%, p = 0.04), with an ORadjusted of 3.3 and an ICadjusted ranging from 1.24 to 8.78.

#### Self-esteem among adult prisoners.

Significant differences in self-esteem were observed between the two prison populations. Low self-esteem was more frequent in the male prison population (87.4% vs. 69.1%, p < 0.001). Self-esteem within the normal and high ranges were more prevalent in the female prison population (20.6% vs. 8.9%, p < 0.001 and 10.3% vs. 3.6%, p = 0.002, respectively).

### Criminal history among adult prisoners

A higher proportion of individuals from the male prison were incarcerated for serious offenses, and also a higher proportion had previously been incarcerated (42.95%). In addition, participants from the male prison reported a younger mean age at first-time incarceration 21 years [18–29] vs 28 years [21–37] for those from the female prison (p < 0.001). Table 4 shows more details.

**Table 4:** Distribution of reported types of crime and recidivism between two populations with different gender compositions.

	Males n (%)	Females n (%)	p-value	OR	ORa*	ICa*
Felony (n = 568) Yes No	178 (**44.2%)** 225 (55.8%)	22 (13.2%) 143 (86.7%)	**<0.001**	**5.14**	**4.71**	**[2.83-7.84]**
Misdemeanor offenses (n = 568) Yes No	131 (32.5%) 272 (67.5%)	59 (35.8%) 106 (64.2%)	0.456	**_**		**_**
Infractions (n = 568) Yes No	5 (1.2%) 398 (98.8%)	2 (1.2%) 163 (98.8%)	1.000	**_**		**_**

* Adjustment for age, educational level, marital status, recidivism and gender.

## Discussion

This study highlighted differences in mental health issues among adult prisoners in two Tunisian jails with different gender compositions. More specifically, during childhood, physical abuse was more common among the male prison population, while sexual abuse was four times more frequent among the female prison population. Participants in the male prison reported greater exposure to childhood extra-familial adversities. Substance use was more prevalent among participants in the male prison across all substance types assessed. Regarding mental health, the female prison population experienced more anxiety, depression, suicidal ideation, and poor sleep quality, while low self-esteem was more prevalent among the male prison population.

In terms of **childhood trauma**, the average total ACE score was significantly higher for the male prison population (4.98 ± 1.94 vs 4.5 ± 2.48). **Regarding intra-familial ACEs**, physical abuse was significantly more common among the male prison population, while sexual abuse was five times more frequent among the female prison population. This finding replicates a consistent finding in the literature that females experienced sexually- and emotionally-related abuse as children [10,11]. Concerning physical abuse, our results align with a study conducted among adolescents from the Fragile Families and Child Wellbeing Study, which showed that 62.9% of males were victims (vs. 57.4%) [30]. Furthermore, we found a higher prevalence of physical abuse among the male prison population. Research attributed this finding to socialization patterns like physical toughness and greater exposure to violence [31]. Indeed, our cultures cultivate specific behaviors and expectations for boys. For example, boys are often encouraged to hide their vulnerability, participate in physically demanding activities, and demonstrate resilience. On the other hand, girls may be more vulnerable to sexual exploitation and abuse during childhood due to societal gender norms [32]. They are perceived as passive individuals and victims of power imbalances. In Tunisia, about 14% of women reported having been sexually abused by an intimate partner during their lifetime and 10.7% were victims of sexual violence consisting mainly of rape and sodomy, at least once in their lifetime [33]. **Regarding social ACEs**, the male prison population was five times more likely to experience at least one social ACE. This population reported significantly more exposure to community and collective violence. Our result aligns with a Tunisian study performed among adolescents [34,35]. Parents in our culture often give boys more freedom to spend time outside the home, making them more vulnerable to harassment, sexual abuse, and community violence than girls [23]. In contrast, girls are typically treated as fragile and protected from such violent experiences outside the home [23]. However, our design does not allow us to isolate the role of gender.

**Concerning substance use among prisoners**, our findings reveal that **tobacco use** was significantly higher among the male prison population (76.9% vs 55.8%; p < 0.001). These data align with international trends. But Tunisian figures were slightly higher than those reported in Spain (74.4% males vs 67.4% females) [36] or Zambia (51.6% males vs 43.9% females; OR=16 [5.1–53.0]) [37]. Another noteworthy finding concerns the significantly higher prevalence of severe nicotine dependence among the female prison population. The paradox of lower consumption yet higher dependence aligns with existing literature. It may reflect compensatory tobacco use to cope with incarceration-related stress, with prison conditions exacerbating addiction, thus increasing female vulnerability [38]. In our study, **alcohol consumption** was significantly higher among the male prison population than among the female prison population, a trend also observed for **illicit drug use**. Notably, our findings contrast with studies from high-income countries, which often report higher rates of drug-related disorders among female prisoners [39,40]. This discrepancy may reflect sociocultural specificities in Tunisia, where female incarceration remains rare and frequently linked to high-risk profiles [39,41]. Furthermore, we found differences in the physiological effects of substances due to pharmacokinetic properties and sensitivity to gonadal hormones, with females being intoxicated with low doses of alcohol [18].

**In terms of mental health issues, anxiety and depression** were more common in the female prison population than in the male prison population, and they also experienced more severe symptoms. Our results align with a systematic review [42]. Other studies conducted among prisoners in South of Brazil [43], Iran [44] and Ethiopia [45], showed a high prevalence of anxiety and depression among female prisoners. Hormonal fluctuations may increase vulnerability to anxiety and depression, along with a history of childhood trauma, such as sexual abuse, which was common among our female prison population [43]. Previous research has suggested that stress affects the frontal cortex differently in males and females, resulting in different responses in the glutamate, GABA, and BDNF systems [13]. **Suicidal thoughts** during incarceration in our sample were significantly higher among the female prison population. A 2022 systematic review and meta-analysis found that both men and women face elevated suicide risks post-incarceration, with women at a notably higher risk [46]. Additionally, non-suicidal self-injury, in-prison drug use, and severe psychological distress have been identified as factors associated with recent suicidal thoughts among these females [47]. In contrast, **self-harming behaviors** were reported at similar rates among our male and female prison population (45.9% vs 43%; p = 0.535). Self-harming behavior is a pervasive problem in jails and prisons with research estimates indicating that about 30% of prisoners engage in the behavior [48]. Tunisian research conducted among male prisoners showed that childhood collective and peer violence predict self-directed violence through recent exposure to violence [19].

However, our results showed that the female prison population was significantly more likely to engage in self-starving, self-hitting and intentional medication overdose compared to the male prison population. Research suggested that women in prison engage in self-harm through behaviors associated with psychological distress and control, whereas men may exhibit different coping mechanisms. In terms of **sleep disorders**, our study found a high prevalence of poor sleep disorders among prisoners, with the female prison population being more likely to have poor sleep quality. A 2022 systematic review highlighted that environmental factors inherent to prison life contribute significantly to sleep disturbances, affecting both male and female prisoners [49]. These environmental factors include noise, overcrowding, light, and temperature. However, our results revealed a significant difference in **self-esteem**, with the male prison population being more likely to report low self-esteem compared to the female prison population. This may be related to societal expectations, where masculinity is often associated with autonomy and control. The loss of freedom and social status in prison may therefore have a more profound impact on self-esteem among males [50]. Further research is needed to explore why male prisoners may be vulnerable to low self-esteem and to identify the factors contributing to this specific effect.

**Regarding criminal history**, the male prison population had a younger age at first-time incarceration [51]. Felonies rates were significantly higher among the male prison population than the female prison population. This result coincides with other studies regarding the differences between males and females in criminal behaviors [52–54]. In fact, it was proven that experiencing adverse childhood experiences is associated with higher rates of criminal behaviors, particularly physical and sexual abuse, as a predictor of reoffending [55]. Furthermore, substance abuse history was highly prevalent among our male prison population, contributing to increased criminality. Besides, while intoxicated men commit the index offense [53]. Moreover, elevated testosterone levels are linked to increased criminal behaviors [56]. A recent study published on testosterone levels among male rapists in Enugu State revealed that the testosterone concentrations in violent rapists, non-violent rapists, and violent child molesters were higher than those in non-rapists [57].

This study has some limitations to be acknowledged. Firstly, the number of participants from the female prison was lower than that of the male prison population. Limited sample size, reducing statistical power. However, it was due to limited accessibility. We made every effort to include the maximum number of adult prisoners. Second, the data were based on self-reported information, which may be subject to bias. For instance, given the retrospective nature of the questions, recalling childhood trauma or mental health issues may lead to underreporting of experiences. However, we used Arabic-validated tools to enhance the accuracy and validity of the responses. Social desirability and fear of repercussions may also suppress reporting of drug use, suicidality, and ACEs, especially among illiterate prisoners who were assisted by an investigator, which may be associated with information bias. But data collection was conducted in a private setting, away from other participants, and each participant was interviewed individually by trained investigators. In addition, participants were required to sign the questionnaire without providing any identifying information. In prison, this requirement may have affected the disclosure of sensitive information, leading to underreporting of some experiences and behaviors. However, participants were explicitly informed that responses would remain strictly confidential and anonymized and that access to the data was restricted to the research investigators only. Third, substance use and suicidality were assessed through a yes or no question, instead of a validated tool to assess frequency, duration, or severity of the behavior, thereby limiting the granularity of the findings. This choice was made to simplify the questionnaire and keep it concise.

Another limitation is that participants were recruited from only two prisons located in different regions of Tunisia. Hence, we can not generalize our results at the national level. Additionally, data collection was conducted at different times and in two prisons that differed in gender compositions, as well as prison settings, which is associated with bias. But it was due to logistical and administrative authorization constraints, as simultaneous access to multiple prisons was not feasible. Consequently, gender is confounded with prison, region, and survey wave, which limits comparability and causal interpretation. Despite multivariable adjustment, the cross-sectional design limits causal inference; thus, findings should be interpreted as associations, and future intervention studies are needed to establish causality or the explanatory role of gender. Despite adjustments, residual confounding could remain due to prison-specific characteristics or shifts in national economic conditions over time.

Additional limitations to be acknowledged include possible volunteer bias (non-participants may have different characteristics) and important unmeasured confounders such as psychiatric diagnoses, psychotropic medications, and prison overcrowding. We have to

Finally, further research should be conducted among Tunisian jail‐based populations to help improve the current gaps. But we believe the strengths of this initial study and the importance of the findings outweigh the limitations.

## Conclusion

Our survey highlighted the differences in mental health issues among adult prisoners in two Tunisian jails with different gender compositions. These findings suggest the importance of implementing strategies and interventions. Treating mental health issues and substance abuse among incarcerated individuals has been consistently associated in the literature with improved post-release outcomes. Providing access to appropriate therapy, counseling, and rehabilitation programs has also been suggested to be more cost-effective than incarceration and may help mitigate negative outcomes, including reoffending rates.
